# SmartTracing: self-learning-based Neuron reconstruction

**DOI:** 10.1007/s40708-015-0018-y

**Published:** 2015-08-19

**Authors:** Hanbo Chen, Hang Xiao, Tianming Liu, Hanchuan Peng

**Affiliations:** 1 Allen Institute for Brain Science, Seattle, WA USA; 2 Cortical Architecture Imaging and Discovery Lab, Department of Computer Science and Bioimaging Research Center, The University of Georgia, Athens, GA USA; 3 CAS-MPG Partner Institute for Computational Biology, Shanghai Institutes for Biological Sciences, Chinese Academy of Sciences, 320 Yueyang Road, Shanghai, China

**Keywords:** SmartTracing, Neuron reconstruction, Neuron morphology, Machine learning, Reconstruction confidence

## Abstract

In this work, we propose SmartTracing, an automatic tracing framework that does not require substantial human intervention. There are two major novelties in SmartTracing. First, given an input image, SmartTracing invokes a user-provided existing neuron tracing method to produce an initial neuron reconstruction, from which the likelihood of every neuron reconstruction unit is estimated. This likelihood serves as a confidence score to identify reliable regions in a neuron reconstruction. With this score, SmartTracing automatically identifies reliable portions of a neuron reconstruction generated by some existing neuron tracing algorithms, without human intervention. These reliable regions are used as training exemplars. Second, from the training exemplars the most characteristic wavelet features are automatically selected and used in a machine learning framework to predict all image areas that most probably contain neuron signal. Since the training samples and their most characterizing features are selected from each individual image, the whole process is automatically adaptive to different images. Notably, SmartTracing can improve the performance of an existing automatic tracing method. In our experiment, with SmartTracing we have successfully reconstructed complete neuron morphology of 120 *Drosophila* neurons. In the future, the performance of SmartTracing will be tested in the BigNeuron project (bigneuron.org). It may lead to more advanced tracing algorithms and increase the throughput of neuron morphology-related studies.

## Introduction

The manual reconstruction of a neuron’s morphology has been in practice for one century now since the time of Ramón y Cajal. Today, the technique has evolved such that researchers can quantitatively trace neuron morphologies in 3D with the help of computers. As a quantitative description of neuron morphology, the digital representation has been widely applied in the tasks of modern neuroscience studies [1–3] such as characterizing and classifying neuron phenotype or modeling and simulating electrophysiology behavior of neurons. However, many popular neuron reconstruction tools such as Neurolucida (http://www.mbfbioscience.com/neurolucida) still rely on manual tracing to reconstruct neuron morphology, which limits the throughput of analyzing neuron morphology.

In the past decade, many efforts have been given to eliminate such a bottleneck by developing automatic or semi-automatic neuron reconstruction algorithms [1, 3]. In these algorithms, different strategies and models were applied, such as pruning of over-complete neuron trees [4, 5], shortest path graph [6], distance transforms [7], snake curve [8], and deformable curve [9]. However, the completeness and the attribute of resulted neuron morphology vary tremendously between different algorithms. Recently, to quantitatively assess such variability between algorithms and advance the state of the art of automatic neuron reconstruction method, a project named BigNeuron [10, 11] has been launched to bench-test existing algorithms on big dataset. One reason causing such variability is that image quality and attributes vary between different data sets—partially due to the differences in imaging modality, imaging parameter, animal model, neuron type, tissue processing protocol, and the proficiency of microscopic operator. And some of the algorithms were developed based on specific data or were developed to solve specific problem in the data which may not be applicable for other types of data. Another reason is that most of the tracing algorithms required user input of parameters. As a consequence, the optimal parameters vary between images and thus require manual tuning by the user with sufficient knowledge of the algorithm.

We note that most of the current automatic neuron reconstruction algorithms are not “smart” enough. Indeed, many times they require human intervention to obtain reasonable result. To conquer this limitation, one can adapt learning-based methods; so the algorithm can be trained for different data. In [12], the authors proposed a machine learning approach to estimate the optimal solution of linking neuron fragments. However, the fragments to link were still generated by model-driven approaches, and it requires manual work in generating training samples.

**Fig. 1:**
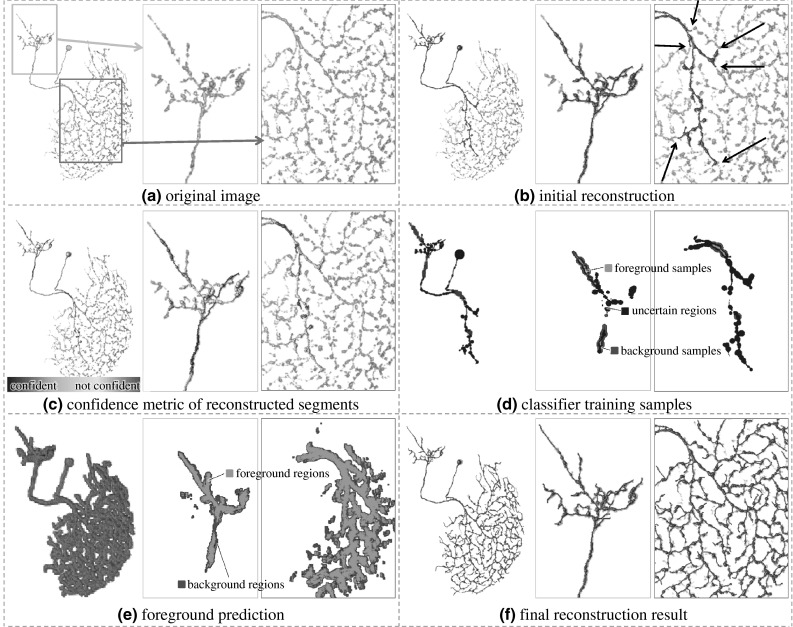
Overview of SmartTracing method and the result for a single image. In each sub-figure, the global 3D view of images and the overlapped reconstructions is shown on the *left*. The zoomed-in 3D view (**a**–**c**) and (**f**) or slice view (**d**–**e**) are shown on the *right*. The locations of the zoomed-in view are *highlighted* in **a**

In this paper, based on machine learning algorithms, we proposed SmartTracing, an automatic tracing framework that does not require human intervention. The procedure of the SmartTracing algorithm is outlined in Fig. 1. First, the initial reconstruction was obtained based on existing automatic tracing algorithms (Fig. 1b). Second, a confidence metric proposed in this paper was computed for each reconstruction segment to identify reliable tracing (Fig. 1c). Third, a training sampler (Fig. 1d) and the most characteristic features were obtained. Fourth, a classifier was then trained and the foreground containing neuron morphology was predicted (Fig. 1e). Finally, after adjusting the image based on prediction result, the final reconstruction was traced (Fig. 1f).

The paper is organized as follows. We first discuss the key steps of SmartTracing. Then we describe the implementation and the availability of the algorithm. Finally, we present experimental results on real neuron image data, followed by some brief discussion of the pros and cons and the future extension of SmartTracing.

## Method

### Automatic search training exemplars

#### Confidence score of reconstruction

**Fig. 2:**
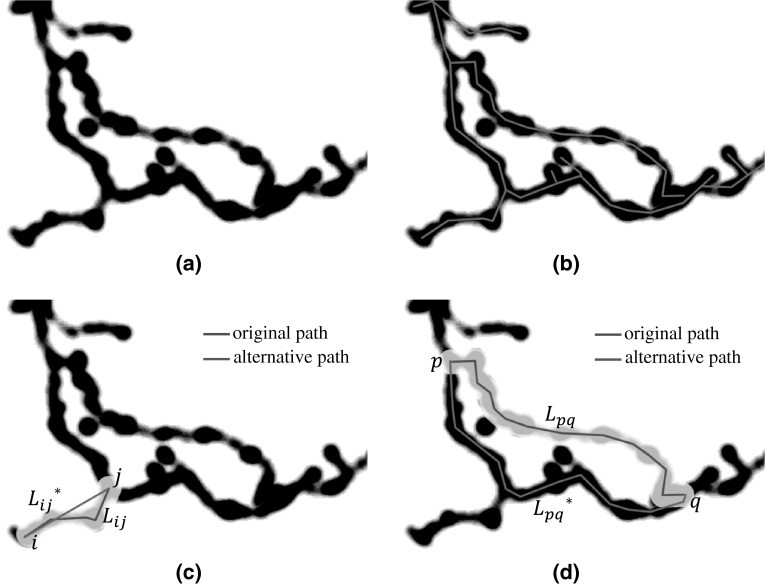
Illustration of alternative path. For each segment in the reconstructions, after masking the image along the segment, the alternative path will be searched by fast marching from one end to the other end of the segment based on intensity. **a** neuron to reconstruct, **b** initial reconstructions, **c** alternative path of *L*_*ij*_, **d** alternative path of *L*_*pq*_

In SmartTracing, we first identify the reliable neuron reconstructions as training exemplars. A neuron reconstruction can be decomposed into multiple segments by breaking the reconstruction at the branch point. Whether or not a segment is trustworthy can be tested by checking if there is an alternative path connecting the two ends of the segment compared to this segment. Our premise is that a segment with no better alternative pathway (e.g., Figure 2c) is more reliable in comparison with a segment with alternative pathway (e.g., Figure 2d). Specifically, for a segment *L*_*ij*_ between points *i* and *j*, the image intensity along *L*_*ij*_ will be masked to 0 first. Then, the shortest path $L_{ij}^{*}$ weighted by intensity between points *i* and *j* will be identified. In the original image, the average intensity along *L*_*ij*_ and $L_{ij}^{*}$ will be measured:

1 $$\overline{{I_{ij} }} = \frac{{\int_{{L_{ij} }} {I(x){\text{d}}x} }}{{L_{ij} }},$$

where *I*(*x*) is the intensity of *x* and *L*_*ij*_ is the length of *L*_*ij*_.

Then the confidence metric can be obtained by dividing $\overline{{I_{ij} }}^{*}$ by $\overline{{I_{ij} }}$:

2 $$C_{ij} = \overline{{I_{ij} }}^{*} /\overline{{I_{ij} }}.$$

Our method is that if an alternative path exists, $\overline{{I_{ij} }}^{*}$ will be closer to or even larger than $\overline{{I_{ij} }}$ and *C*_*ij*_ will be close to 1. Otherwise, $L_{ij}^{*}$ will be a relatively straight line passing through background with low intensity connecting *i* and *j*, and thus $C_{ij} \ll 1$. This measurement is based on the assumption that background intensity is lower than foreground intensity. When the background intensity is greater than foreground (e.g., for brightfield images), we can simply invert *C*_*ij*_ in Eq. (2).

#### Obtaining training exemplars

Based on the confidence score obtained, the original image can be classified into 4 groups of regions—foreground samples (labeled neurons), background samples (no-neuron area), uncertain regions, and the irrelevant area (Fig. 1d). Foreground samples are defined as the skeleton regions of confident reconstruction segments. Background samples are defined as the non-skeleton regions surrounding the confident reconstruction segments. The intermediate zones between these two regions are taken as uncertain regions. And the zones surrounding less-confident reconstructions are taken as uncertain regions as well. These 3 types of regions compose 3 layers surrounding the confident reconstructions—core layer: foreground samples; middle layer: uncertain regions; and outer layer: background samples.

### Extracting features for classification

**Fig. 3:**
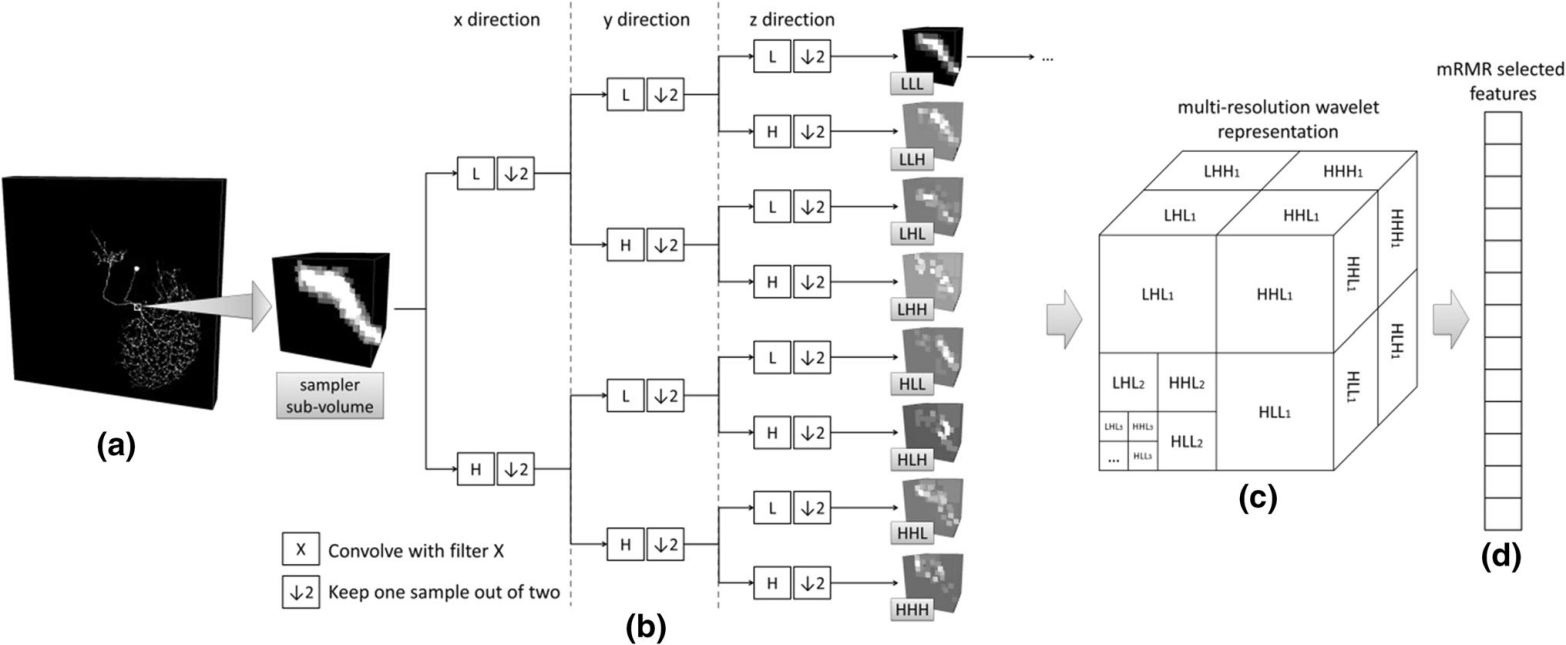
Illustration of feature selection procedure. **a** Extracting sub-volume in 3D cube surrounding the sample voxel. **b** Wavelet decomposition for volume data. **c** Multi-resolution wavelet representation. **d** Selecting a characterizing subset of features based on mRMR for classification

Image intensity-based features are extracted by adopting the method proposed in [13]. The whole procedure is outlined in Fig. 3. For each sample voxel, features are extracted in a 3D cube surrounding this voxel (Fig. 3a). Multi-resolution wavelet representation (MWR) is applied to project the sub-volume of the local 3D cube into a feature space (Fig. 3b. c). Then, a subset of features is selected based on minimum-Redundancy Maximum-Relevance (mRMR) method [14] for classification (Fig. 3d).

MWR codes the information in both frequency domain and spatial domain. It is effective for identifying local and multi-scale features from signals or images and has been widely used in pattern recognition tasks. The MWR framework was firstly introduced on 1-dimensional (1D) signals and then extended to 2-dimensional (2D) images by Mallat [15]. In brief, a pair of functions was defined to conduct wavelet transform—the mother wavelet *ψ*(*x*)—representing the detail and high-frequency parts of a signal and the scaling function *φ*(*x*) representing the smooth and low-frequency parts of the signal. To decompose signal into multiple resolutions, the calculation is performed iteratively on the smoothed signal calculated based on *φ*(*x*). In practice, for discrete signal, instead of calculating wavelet *ψ*(*x*) and scaling function *φ*(*x*), a high pass filter H and a low pass filter L will be applied to calculate MWR. Mallat has shown that MWR can be extended from 1D signal to 2D image by convolving the image with the filters in one dimension first and then convolving the output image with the filters in the other dimension [15]. Such operation can be further extended to 3D volume [16]. As illustrated in Fig. 3b, in one level of decomposition, 8 groups of wavelet coefficients are obtained by convolving volume with different permutations of two filters in three directions successively. The smoothed volume LLL is further decomposed in the next level to achieve multi-resolution representations.

After MWR decomposition, the dimension of feature space is relatively high—the number of features $\{ f_{i} \}$ equals the number of voxels in the sub-volume (Fig. 3c). Since some of these features may carry redundant information or non-discriminative information, using the full set of MWR coefficients directly may lead to inaccurate result. To better discriminate patterns and improve the robustness and accuracy of training framework, we select the most characterizing subset of features *S*. We consider the mRMR feature selection method to solve the problem. The algorithm has been widely applied in selecting features in high-dimensional data such as microarray gene expression data to solve classification problems [17]. In the algorithm, the statistical dependency between the exemplar type and the joint distribution of the selected features will be maximized. To meet this criterion, mRMR method search for the features that are mutually far away from each other (minimum redundancy) but also individually most similar to the distribution of sampler types (maximum relevance). In practice, these two conditions were optimized simultaneously:

3 $$\mathop {\hbox{max} }\limits_{S \in W} \left\{ {\frac{1}{\left| S \right|}\mathop \sum \limits_{i \in S} I(c,f_{i} ) - \frac{1}{{\left| S \right|^{2} }}\mathop \sum \limits_{i,j \in S} I(f_{i} ,f_{j} )} \right\},$$

where *W* denotes the full set of MWR coefficients, *c* denotes the vector of sampler type, $\left| S \right|$ is the number of features, and *I*(*x*, *y*) is the mutual information between *x* and *y*. The first term in the equation is the maximum relevance condition, and the second term is the minimum redundancy condition. It has been shown in [14] that the solution can be computed efficiently in $O(\left| S \right|*\left| W \right|)$.

### Training classifier and tracing neuron reconstruction

Based on the extracted features of training samplers, supervised training can be performed to train a classifier for foreground/background predictions. In our proposed framework, we use Support Vector Machine (SVM) implemented in LIBSVM tool kit [18]. The default parameter setting of LIBSVM is used. A subset of foreground and background training samplers is randomly chosen from the pool to make sure that the numbers of training samplers from each class are the same.

With the trained classifier, we then examine the voxels in the image and label them as foreground or background (Fig. 1e). Since in neuron tracing problem foreground signals are often sparse and relatively continuous in the image, we use a fast marching algorithm to search for the foreground signals. Initially, the voxels of foreground samples are pre-labeled as foreground and the rest voxels are marked “unknown.” The algorithm would then march from foreground voxels to their adjacent unknown voxels. For each of such “unknown” voxels, its feature will be extracted and will be classified into foreground or background based on the classifier trained. If the voxel is classified as foreground, it will be taken as a new starting point for the next round of marching. The marching will stop if no more foreground voxel can be reached, and all of the unknown voxels left will be labeled as background.

Based on the labeled image, the original image is adjusted to obtain the final tracing result. The intensity of background voxels is set to 0. For foreground voxel, if its intensity is lower than threshold set for tracing algorithm, the intensity of the voxel will be set as the threshold value. Otherwise, its intensity will be kept unchanged. Then the tracing algorithm will be re-run on the adjusted image to trace the final corrected neuron reconstruction.

## Implementation

Intuitively, the proposed sampling, training, and prediction framework can be applied on any existing neuron tracing algorithms to test and improve its performance. In our implementation, we used the APP2 tracing algorithm [4] to generate the initial tracing from original image as well as the final tracing from the image after prediction. To our best knowledge, APP2 tracing algorithm is the fastest tracing algorithm among existing methods and is reliable in generating tree shape morphology for neuron reconstructions, which makes it an ideal algorithm to implement proposed framework. On the other hand, the APP2 algorithm has its own limitations. It will stop tracing when there is a gap between signals such as the ones highlighted by arrows in Fig. 1b. Also, like many other tracing algorithms, it needs to fine tune the background threshold and other parameters to avoid over-tracing. Thus, our proposed framework can further improve the performance of APP2.

We implemented the SmartTracing algorithm as a plugin of Vaa3D [19, 20] which is the common platform to implement algorithms for the BigNeuron project (bigneuron.org) bench-testing. Since the APP2 algorithm has already been implemented in Vaa3D, the algorithm was directly invoked via the Vaa3D plugin interface. The default parameters of APP2 were taken to generate initial neuron reconstruction. To generate the final reconstruction, the background threshold was set to 1 since the intensity of all the background voxels was set to 0 as introduced in the previous section. The neighborhood 3D window size was 16 × 16 × 16 voxels. The cube of each such 3D small window was decomposed into 3 levels of MWR. The mRMR feature selection was implemented based on the code downloaded from http://penglab.janelia.org/proj/mRMR/, and the top 20 characteristic features were selected. Classifier training and prediction were implemented based on the code downloaded from LIBSVM tool kit (http://www.csie.ntu.edu.tw/%7cjlin/libsvm/).

## Experimental results

**Fig. 4:**
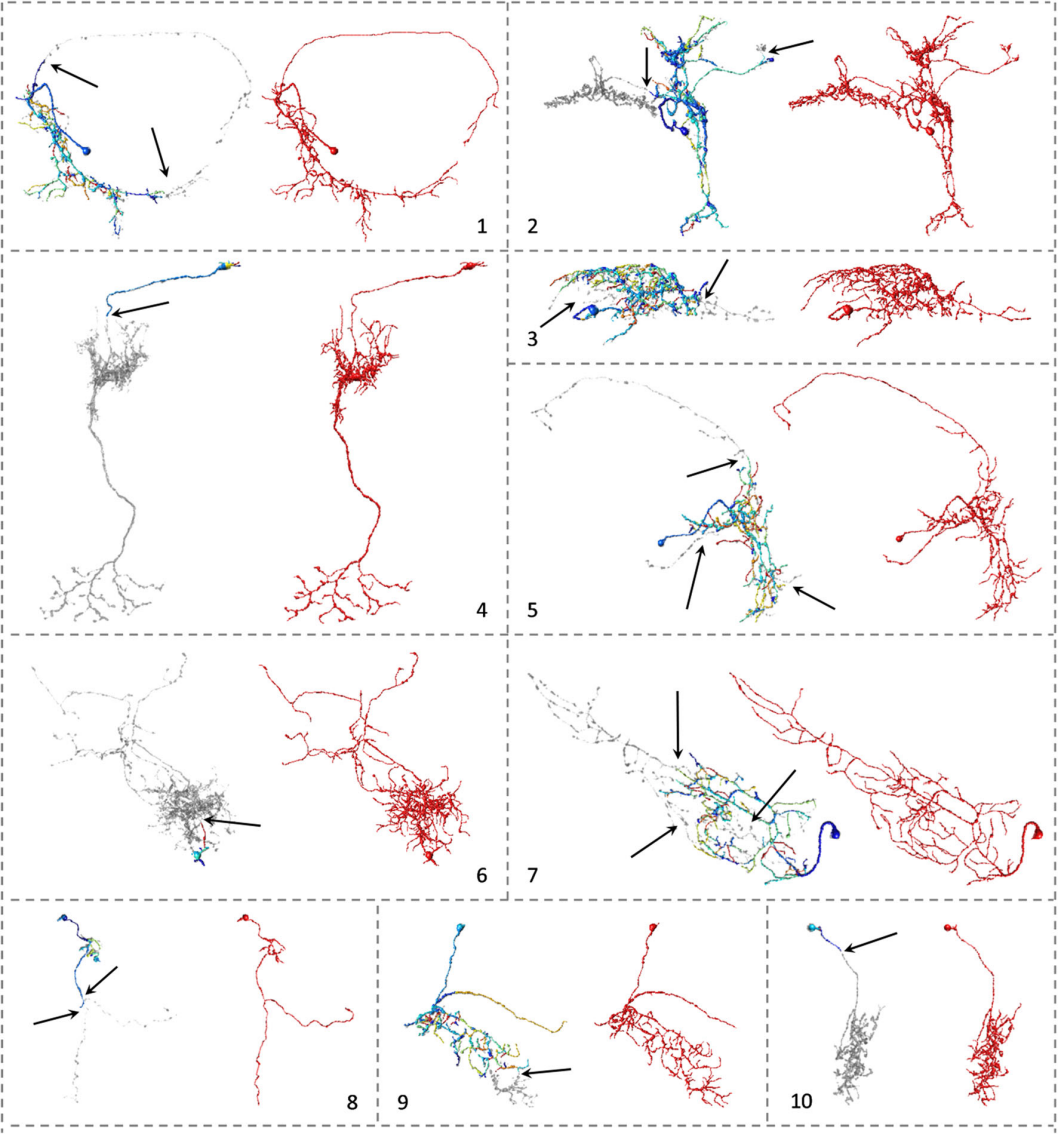
Visualization of reconstructed neuron morphology of 10 selected examples. In each sub-figure, initial reconstruction generated by APP2 (*colored* skeletons) was overlapped on the original image (*gray* skeletons). The corresponding final reconstruction obtained by SmartTracing was shown in *red* skeletons on the *right*. The initial reconstructions were color coded by confidence scores (*blue* more confident, *red* less confident). The incomplete part of the reconstruction and the gap that caused the problem were *highlighted* by *black arrows*. The detailed measurements of these reconstructions are listed in Table 1. (Color figure online)

The whole framework was tested on 120 confocal images of single neurons in the *Drosophila* brain downloaded from the flycircuit.tw database. The dimension of each image is 1024 × 1024 × 120 voxels. For some of the images, APP2 works reasonably well in reconstructing neuron morphologies. However, due to the loss of signals during image preprocessing, there could be a gap between neuron segments which resulted in incomplete reconstructions by APP2. Ten examples of incomplete reconstructions were shown and highlighted by arrows in Fig. 4. Those gaps were classified as foreground with proposed SmartTracing framework and filled for complete tracing (red skeletons in Fig. 4). The quantitative measurements of the morphology and the computational running time (using single CPU) of these 10 examples are listed in Table 1.

**Table 1:** The running time of each procedure and the quantitative neuron morphology measurement of 10 selected example datasets

ID	Running time (seconds)						Length		Bifurcation		Branch		Tip	
	*T*_in_	*T*_s_	*T*_m_	*T*_t_	*T*_p_	*T*_st_	*R*_in_	*R*_st_	*R*_in_	*R*_st_	*R*_in_	*R*_st_	*R*_in_	*R*_st_
1	10.4	254	0.19	12.7	110	12.7	3027	4686	69	74	141	153	72	79
2	10.6	456	0.22	17.9	185	15.1	4469	7557	112	180	228	367	116	187
3	11.1	474	0.23	12.1	89	14.6	4611	6163	145	159	293	325	149	167
4	9.2	310	0.17	7.4	58	15.9	483	5823	5	117	11	240	7	124
5	10.9	310	0.19	8.5	119	16.7	3992	5635	84	92	175	188	91	96
6	9.2	29	0.17	7.5	133	22.2	176	8298	4	174	9	359	6	186
7	9.3	249	0.16	7.9	120	19.2	4408	7016	74	98	151	198	77	101
8	9.3	61	0.17	11.6	69	9.9	545	1174	7	8	14	16	8	9
9	10.1	307	0.17	9.2	53	13.4	3021	4024	75	93	155	190	81	98
10	9.0	37	0.16	7.3	78	15.3	125	3494	2	76	5	159	3	83

Visualization of the morphology of reconstructions and the original image of these examples are shown in Fig. 4

*T*_in_ generating initial reconstruction by APP2; *T*_s_ computing confidence score; *T*_m_ mRMR feature selection; *T*_t_ SVM classifier training; *T*_p_ searching foreground; *T*_st_ generating final reconstruction; *R*_in_ initial reconstruction; *R*_st_ final reconstruction; *Length unit*: voxel

**Fig. 5:**
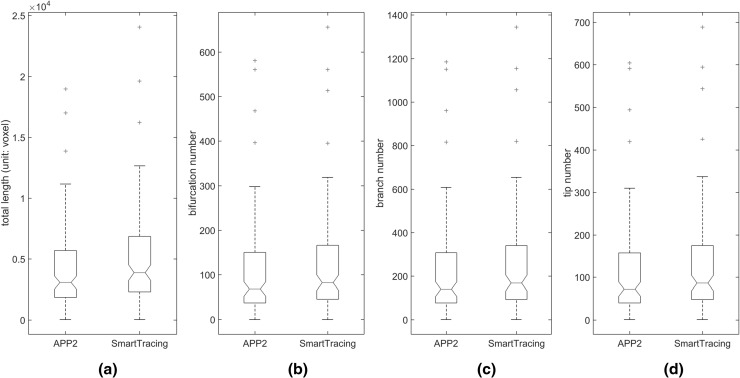
*Box plots* of neuron morphology measurements of the 120 neuron reconstructions obtained

**Fig. 6:**
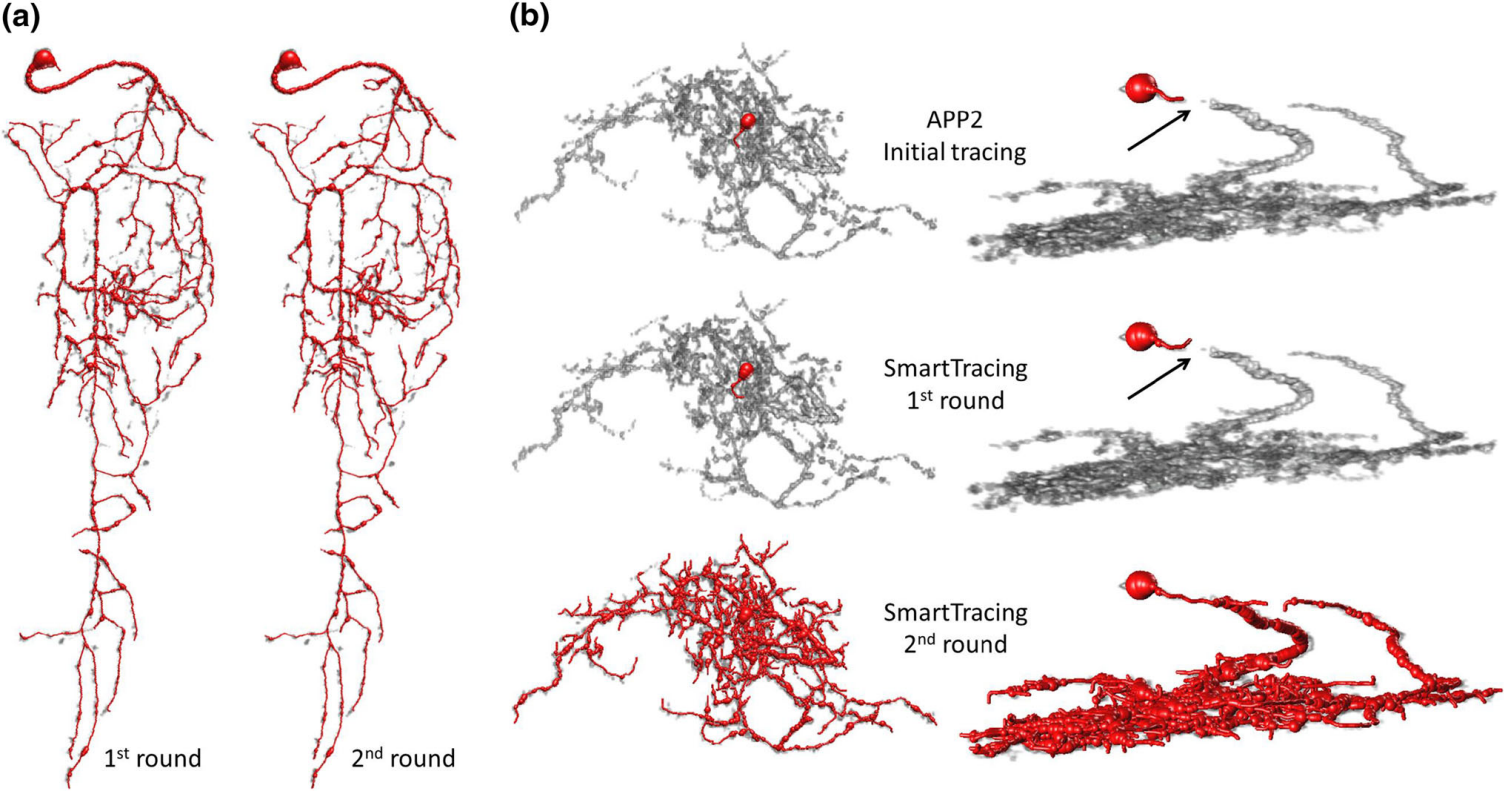
Examples of performing SmartTracing iteratively. Reconstruction shown in *red* tube is overlapped on the original image shown in *gray*. **a** Reconstruction of the first and second rounds of SmartTracing of case #7 shown in Fig. 4. **b** Reconstruction of the case that failed in the first round of SmartTracing but succeeded after two *rounds* shown in different angles. The gap that caused the failure in the first round is *highlighted* by *arrows*. (Color figure online)

For the 120 confocal images tested, the proposed SmartTracing algorithm successfully improved the overall completeness of reconstructions. In comparison with initial reconstructions, the total length, bifurcation number, branch number, and tip number all increased after the optimization of SmartTracing (Fig. 5). Among those, the completeness of 30 reconstructions was significantly improved (the total length of final reconstruction is 1.2 times larger than that of initial reconstruction). By visual inspection, the SmartTracing algorithm only failed to trace the complete neuron morphology on 1 image out of the 120 images. In this failure case, there is a gap that is too big to be filled (Fig. 6b).

Notably, SmartTracing is able to run iteratively. The reconstruction generated from the previous round is used as the initial reconstruction for the next round. However, for the reconstruction that is relatively complete, further iteration will not change the result significantly (Fig. 6a) and is time consuming. On the other hand, for the incorrect reconstruction, better training samples could be obtained based on the reconstruction from the previous iteration which may successively remedy the reconstruction. Thus we tried performing SmartTracing iteratively on the previously failed case. Intriguingly, it only took two rounds of SmartTracing to successfully fill the gap and obtain complete reconstruction (Fig. 6b). This is mainly because. with the result from the first round, more training samples from the gap area were obtained to train the classifier, so the gap can be filled in the second round.

**Fig. 7:**
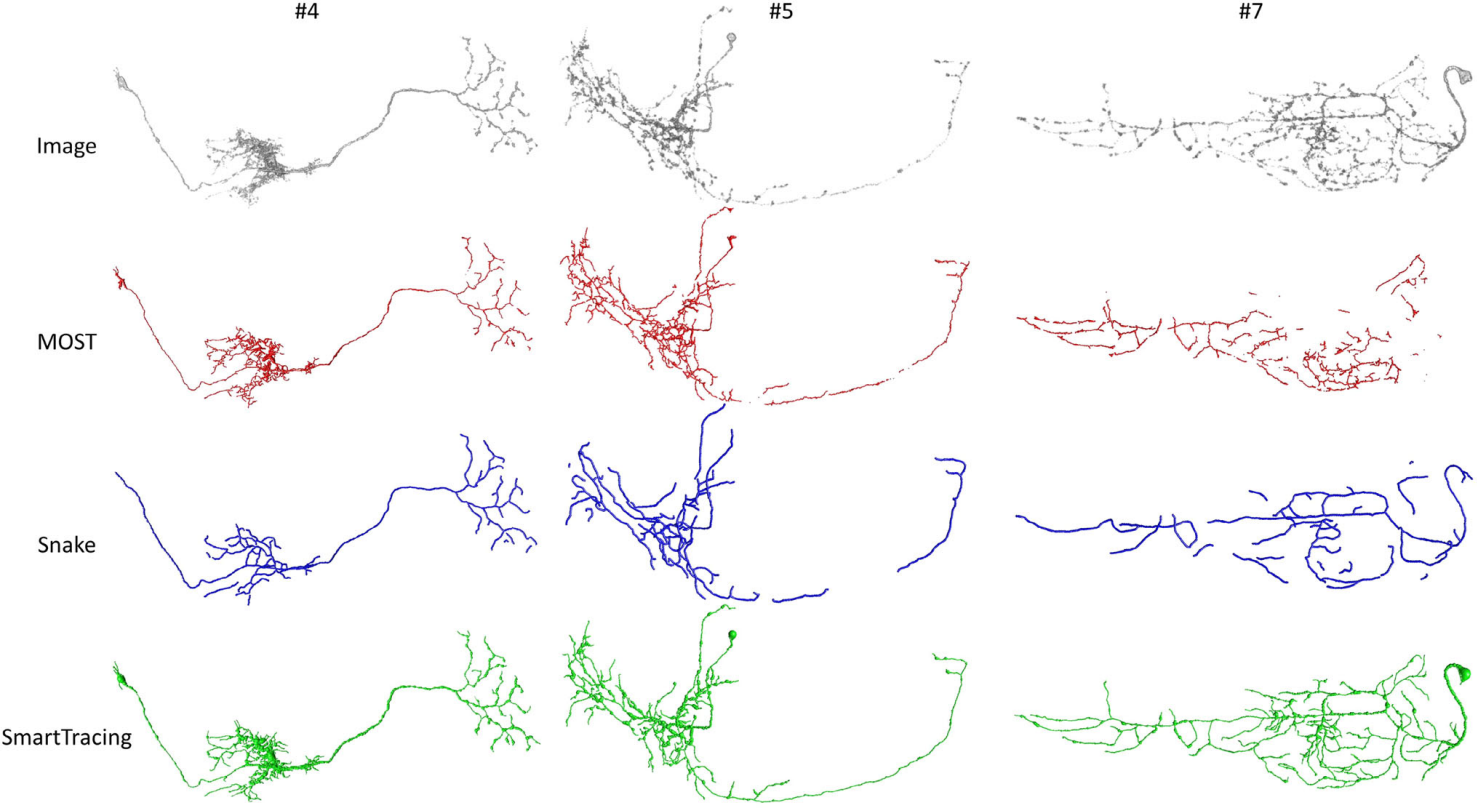
Comparisons of the reconstructions generated by 3 different tracing algorithms using 3 testing images. Image ID is the same as Fig. 4. The original images are shown in the *top row* followed by the reconstructions generated by MOST (*red*), Snake (*blue*), and SmartTracing (*green*). (Color figure online)

We then compared the result generated by SmartTracing with other methods. Specifically, the results generated by micro-optical sectioning tomography (MOST) ray-shooting tracing [21] and open-curve snake (Snake) tracing [8, 22] were compared. By visual inspection, the results generated by our proposed SmartTracing were more complete, more topologically correct, and better at reflecting the morphology of the neurons in original images than other tracing methods (Fig. 7).

## Discussion

In our experiments, the proposed SmartTracing method improved the APP2 tracing and successfully reconstructed 120 *Drosophila* neurons from confocal images. In addition to filling the gaps between neuron segments, SmartTracing can also reduce over-traces due to image noise, inhomogeneous distribution of image intensity, and inappropriate tracing parameters. Essentially, SmartTracing is an adaptive and self-training image preprocessing procedure that segments the image into the foreground area containing neuron signals and the background voxels. The major novelty of SmartTracing lies in two aspects.

First, we proposed a likelihood measurement that serves as a confidence score to identify reliable regions in a neuron reconstruction. With this score, reliable portions of a neuron reconstruction generated by some existing neuron tracing algorithms are identified, without human intervention, as training exemplars for learning-based tracing method. On the other hand, the human proofreader can also benefit from the metric. By ranking the reconstructions by the confidence score, the human annotators are able to prioritize on the less-reliable reconstructions, which increases the overall accuracy and saves time.

Second, from the training exemplars the most characteristic wavelet features are automatically selected and used in a machine learning framework to predict all image areas that most probably contain neuron signal. Since the training samples and their most characterizing features are selected from each individual image, the whole process is automatically adaptive to different images and does not require prior knowledge on the object to identify. Potentially, the proposed machine learning and prediction framework can be extended to other image segmentation tasks and 3D object recognition systems such as neuron spine detection, cell segmentation, etc.

SmartTracing is applicable to most of the existing tracing algorithms. However, the performance and the outcome of SmartTracing largely relied on the tracing algorithm applied. For instance, the cause of the only failed case among 120 tested images is that APP2 did not generate sufficient initial reconstruction due to the gap which results in a lack of training exemplars. One solution to this limitation is to run SmartTracing iteratively, so better training samples can be acquired from the previous iteration. Also, we can take the merit of different tracing algorithms and use different algorithms in different steps to further improve the performance of the framework—e.g., use MOST algorithm to generate initial tracing for scoring and thus training since it is not sensitive to gaps and can capture more signals; then use APP2 to generate final tracing since it is robust, efficient, and optimal to generate tree shape topology of neurons.

Another limitation of SmartTracing is the relatively high computational complexity. At present, the top two time-consuming procedures are the computation of confidence metric, which is proportional to the initial neuron reconstruction complexity, and the predictions of foreground voxels, which is proportional to the size of the neuron. The previously reported computation time is calculated based on a single CPU. With parallel computation framework, both steps can be sped up.

In recent years, a growing number of model-driven approaches have been proposed for automatic neuron reconstructions. To our best knowledge, SmartTracing is one of the earliest machine learning-based methods for automatic neuron reconstruction. Different from the traditional learning-based method, SmartTracing does not require human input of training exemplars and can self-adapt to different types of neuroimage data. Additionally, the method can be applied to improve the performance of other existing tracing methods. As part of future work, the performance of SmartTracing will be further examined and improved by BigNeuron project. In the near future, we hope that SmartTracing can significantly facilitate manual tracing and contribute to the neuron morphology reconstructions in large.
